# Extracorporeal membrane oxygenation before and after adult liver transplantation: worth the effort?

**DOI:** 10.1186/cc13393

**Published:** 2014-03-17

**Authors:** G Auzinger, C Willars, R Loveridge, A Vercueil, T Best, J Wendon

**Affiliations:** 1 King's College Hospital London, UK

## Introduction

Extracorporeal membrane oxygenation (ECMO) is increasingly used for the treatment of refractory but potentially reversible respiratory and/or cardiac failure. Data on perioperative support with veno-arterial (V-A) and veno-venous (V-V) ECMO for adult liver transplant recipients are scarce [1,2].We report our experience of ECMO support in patients with acute liver failure (ALF) as a bridge to transplant and postoperative ECMO use following complications after surgery.

## Methods

A retrospective study in a specialist tertiary referral ICU. Patients supported with V-V or V-A ECMO before, during, or after orthotopic liver transplant (OLT) were identified.

## Results

In total, four patients were supported during a 12-month period. Two patients required V-V and two patients V-A support. Two patients with ALF were bridged to OLT, one patient V-V ECMO for refractory respiratory failure and the other patient required emergency V-A support for treatment of intraoperative arrest. Both patients were successfully transplanted but died subsequently on ECMO: disseminated aspergillosis and haemophagocytic syndrome, and anoxic brain injury respectively. Two patients received postoperative ECMO support. The first was treated with V-V ECMO for refractory persistent hypoxaemia following OLT for hepato-pulmonary syndrome, the second received emergency V-A support (eCPR) following cardiac tamponade and arrest on postoperative day 2. Both patients made a full recovery.

## Conclusion

Emergency ECMO support before and after liver transplant is feasible. Despite the poor outcome in patients with ALF, we consider ECMO a valuable option to bridge selected patients to transplant.
